# Macadamia Carpel Pyrolysis Catalyzed by Natural and Modified Clinoptilolite in a Fixed-Bed Reactor

**DOI:** 10.1021/acsomega.5c00369

**Published:** 2025-05-14

**Authors:** Kelly Costa Cabral Salazar Ramos Moreira, Érica Victor de Faria, Marcelo Antonio de Oliveira, Jesuína Cássia Santiago de Araujo, Kássia Graciele dos Santos, Thiago Padovani Xavier, Taisa Shimosakai de Lira

**Affiliations:** † Programa de Pós-Graduação em Energia, 680695 Universidade Federal do Espírito Santo, Rod. BR 101 Norte, km 60, 29932-540 São Mateus, Espírito Santo, Brasil; ‡ Departamento de Ciências da Saúde, Universidade Federal do Espírito Santo, 29932-540 São Mateus, Espírito Santo, Brazil; § Departamento de Engenharias e Tecnologia, Universidade Federal do Espírito Santo, Rod. BR 101 Norte, km 60, 29932-540 São Mateus, Espírito Santo, Brasil; ∥ Departamento de Engenharia Química, 74348 Universidade Federal do Triângulo Mineiro, R. Frei Paulino, 30-Nossa Sra. da Abadia, 38025-180 Uberaba, Minas Gerais, Brasil

## Abstract

The macadamia carpel, an agro-industrial byproduct, can be valorized as a feedstock for bio-oil production via pyrolysis. However, bio-oils derived from lignocellulosic biomass often exhibit a high oxygen content, which reduces their fuel quality. In this study, natural clinoptilolite (NC) and its modified form (modified clinoptilolite (MC)) were evaluated as catalysts in a fixed-bed pyrolysis reactor to enhance both the liquid yield and the bio-oil quality. The MC catalyst was prepared via ion exchange using 1.0 M NH_4_Cl, followed by calcination at 773.15 K, which improved its textural properties by increasing the surface area from 24.99 m^2^/g to 101.11 m^2^/g, the pore volume from 0.088 m^3^/g to 0.135 m^3^/g, and the average pore diameter from 9.05 to 10.67 nm. Notably, this study is the first to systematically compare macadamia carpel pyrolysis under noncatalytic conditions using both NC and MC, with a focus on enhancing bio-oil quality. Three full factorial experimental designs were conducted, considering reaction temperatures (773.15, 873.15, and 973.15 K); heating rates (10, 30, and 50 K/min); and catalyst concentrations (0, 10, and 20 wt %). Desirability function analysis identified the optimal conditions as a reaction temperature of 873.15 K, a heating rate of 50 K/min, and a catalyst concentration of 20 wt % MC, resulting in a maximum liquid yield of 58.20%. Moreover, GC–MS analysis revealed that the use of MC significantly enhanced the production of phenolic compounds, while reducing undesired oxygenated species. These findings demonstrate that MC improves not only the yield but also the quality of bio-oil, offering a promising strategy for the valorization of macadamia carpel waste.

## Graphical abstract

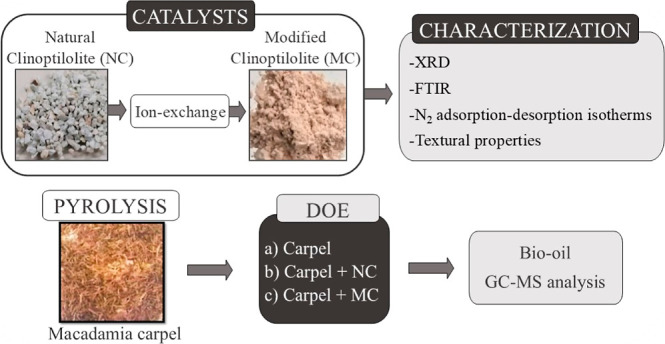

## 1 Introduction

The thermochemical conversion by pyrolysis has been investigated to be applied in organic residues, and several studies show that is an efficient and practical way for biomass conversion into valuable chemicals, biofuels, activated carbon, and electricity.^1–6^ Lignocellulosic biomass, one of the largest residues from agricultural, industrial, and municipal waste, yields three different products—solid (biochar), liquid (bio-oil), and gas—upon pyrolysis.^7–9^ Bio-oil, a high-energy-density liquid, is a complex mixture of many different components formed through the cracking and self-polymerization of lignocellulosic macromolecules.^10^ Its properties depend on the biomass composition, reaction conditions, and the presence of catalysts,^11,12^ making it a potential precursor of fuel and fine chemicals.^13^ Maximum liquid yields are typically obtained at moderate temperatures (673–873 K) and heating rates (<100 K min^–1^),^14^ with a short gas residence time.^15^

Numerous biomass sources, such as sugar cane bagasse,^16–18^ brewer’s spent grain,^19,20^ algal,^21,22^ malt waste,^23^ spent coffee grounds,^24^ cartoon packages,^25^ macadamia nut shell,^26,27^ walnut shell,^28–30^ among others, have been subject to pyrolysis investigations. Although char properties from these sources have been displayed to be suitable for several applications, the potential of macadamia carpel pyrolysis and its potential for bio-oil production remains unexplored.

The macadamia carpel is a lignocellulosic waste from the macadamia nut crop, whose main function is nut protection since it is the external layer of the nut.^29,31^ The shell and the carpel represent the greatest amount of residues generated by the process and contain about 80% of volatile matter,^32^ which indicates that their conversion into bio-oil through pyrolysis could be a great alternative for their destination.^30^

Despite the high energy potential of pyrolysis-derived bio-oil, it shows some disadvantages due to the heterogeneous nature of biomass and its composition characteristics. Its quality is often compromised by high oxygen content, which lowers the heating value, increases viscosity, and reduces stability due to oxidative degradation and increased polarity.^33,34^ To overcome these challenges, catalysts have been employed to improve bio-oil quality by reducing oxygenated compounds and increasing liquid yields.^8,11,13,35^ Among them, zeolites have demonstrated a strong ability to catalytically crack pyrolysis vapors and enhancing bio-oil quality, being considered one of the most promising catalysts materials.^11,36–39^

Zeolites are crystalline aluminosilicates that contain tetrahedra of Si and Al atoms surrounded by oxygen atoms. These atoms create lattices that form cavities of different sizes and shapes.^40^ During pyrolysis, their pore selectivity and the acidic sites in their structure enable catalytic conversion of biomass vapors into valuable products through selective deoxygenation, primarily via C–C and C–O–C bound cleavage through hydrogenolysis.^40–43^

Clinoptilolite is an abundant, low-cost zeolite that is environmentally friendly, highly stable, and nontoxic. It has been investigated as a catalyst in pyrolysis for biochar and bio-oil production.^12,44–47^ Its strong adsorption capacity, high ion exchange ability, and great molecular sieving properties result in superior catalytic performance compared to other natural and synthetic zeolites, leading to increased bio-oil yield due to its large pore structures.^44,47^

To further improve its catalytic properties, clinoptilolite can be modified through a variety of approaches,^44^ such as the ion exchange with NH_4_Cl. This process removes native cations (e.g., Na^+^) from clinoptilolite and, upon subsequent calcination, increases the concentration of Brønsted acid sites. The enhanced acidity not only promotes cracking and deoxygenation reactions but also alters the surface characteristics—yielding improved adsorption capacity and increased hydrophobicity—which facilitates interaction with volatile organic compounds during pyrolysis.^44,48^ As demonstrated by Ambrozova et al.,^44^ these modifications lead to more effective catalytic performance and higher-quality bio-oil production. Similarly, Gurevich Messina et al.^34^ reported that modified clinoptilolite (MC) exhibits superior deoxygenation efficiency, further confirming the beneficial impact of such modifications.

The notable reduction in oxygenated compounds observed with MC stems from its enhanced Brønsted acidity and tailored porosity, which collectively favor deoxygenation pathways (e.g., dehydration, decarboxylation, and decarbonylation) over condensation reactions.^34^ Moreover, the removal of alkali metals (e.g., Na^+^) via ion exchange minimizes unwanted catalytic neutralization, while the newly generated H^+^ sites selectively cleave C–O bonds in oxygenated intermediates.^49,50^

Beyond zeolites, alternative catalysts such as metal oxides/basic catalysts (e.g., MgO), natural minerals (e.g., limestone and magnesium carbonate), amphoteric catalysts (e.g., TiO_2_, ZrO_2_, and CeO_2_), and hybrid systems (e.g., metal-modified zeolites and iron-rich materials) offer promising pathways for bio-oil upgrading. These alternatives provide advantages including higher H/C ratios, effective ketonization, cost-effectiveness, and versatile reaction pathways, yet they also present challenges such as increased coke formation, lower selectivity, variable performance, and higher costs.^51^ In contrast, MC remains particularly attractive due to its low cost, environmental compatibility, and effective performance in enhancing deoxygenation.

Despite these advances, few studies have addressed catalytic bio-oil production from pyrolysis using MC, and most publications do not explore its effects on oil quality. To our knowledge, no prior study has compared fixed-bed pyrolysis of macadamia carpel under both noncatalytic and catalytic conditions using natural and MC as catalysts, with a specific focus on enhancing bio-oil quality.

In this study, we address this critical gap by comparing three distinct pyrolysis approaches: thermal pyrolysis, catalytic pyrolysis with natural clinoptilolite (NC), and catalytic pyrolysis with a modified form of clinoptilolite (MC). The work aimed to evaluate the effects of some parameters on the process; therefore, a pyrolysis optimization strategy was adopted to determine the optimum conditions and best combination of the parameters (temperature, heating rate, and catalyst concentration) to achieve higher bio-oil production.^52^ A design of experiments (DoE) was carried out and the results were statistically treated to maximize the information obtained with fewer experiments.^52–54^ Natural and modified catalysts were characterized, and the pyrolysis experimental designs were performed under predetermined values of temperatures, heating rate, and catalyst concentration, which allowed for the statistical analysis for understanding the parameters effects on bio-oil yield. The optimum conditions were estimated by a desirability function. The produced bio-oil composition was analyzed by gas chromatography/mass spectrometry (GC/MS), to evaluate its quality for application as a fuel.

## 2 Material and Methodology

### 2.1 Material

The macadamia carpel used in this study was provided by Cooperativa Agroindustrial dos Produtores de Noz Macadâmia (COOPMAC), in Esprito Santo, Brazil. The detailed methodology and results of the biomass characterization were previously reported by the research group in,^55^ including proximate and ultimate analyses, as shown in Table 1.

**Table 1:** Proximate and Ultimate Analysis for Macadamia Carpel^a^

components			
proximate composition (%)		ultimate elemental (%)	
M	13.19 ± 0.26	C	45.01 ± 0.95
VM	72.40 ± 1.21	O	48.29 ± 1.26
AC	14.31 ± 0.64	H	5.46 ± 0.65
FC	13.29 ± 0.52	N	1.24 ± 0.52
		S	0.80 ± 0.02
		H/C	0.12 ± 0.015
		O/C	1.07 ± 0.036

a Reprinted with permission from Moreira et al. 2022.^55^ Copyright 2021 Canadian Society for Chemical Engineering.

### 2.2 Modification of Clinoptilolite

NC from Celta Brasil, Slovakia, was employed. The MC was obtained by submitting NC to an ion exchange with NH_4_Cl, wherein 25 mL of a 1.0 M NH_4_Cl aqueous solution was used per gram of clinoptilolite. The reaction mixture was carefully stirred at 353.15 K for 24 h under reflux. The acid-treated solid samples were filtered out under suction by repeated washing with distilled water until the total absence of Cl^–^, which was verified with an AgNO_3_ solution. Finally, the material was dried at about 383.15 K for 20 h and calcined at 773.15 K in a muffle furnace for 4 h, in order to decompose NH_4_^+^ into H^+^.^34,56^

#### 2.2.1 Characterization of Catalysts

The X-ray diffraction (XRD) patterns were recorded on a Rigaku Miniflex 600 diffractometer with Cu–Kα radiation at 1.5418 Å.^57^ The analysis was carried out in the 2θ range from 20° to 80° at a rate of 5° min^–1^. The mean crystallite size (*L*) of both samples were estimated using Scherrer’s equation.^58^

1 $$L=\frac{k\cdot \lambda }{\tau \cdot \cos\theta }$$

where *k* is the Scherrer’s constant, a shape factor (typically 0.91, with a range of 0.8–1.2), λ is the X-ray wavelength (Cu Kα = 0.154186 nm), τ is the full width at half-maximum (fwhm) of the diffraction peak, and θ is the Bragg angle.

To compare the catalyst acidity before (NC) and after modification (MC), the pH of the samples was measured by suspending 1 g of each sample in 20 mL of distilled water and letting the suspension boil for about 1 h. Then, the solution was cooled to room temperature and the pH value was determined using a portable Hanna Edge Dedicated pH Meter HI2002.^34^

Fourier-transform infrared spectra were recorded on an Agilent Technologies Carry 300 spectrometer preparing discs of zeolite with KBr and the analysis were performed with a resolution of 4 cm^–1^ from 4000 to 600 cm^–1^.^59^

Textural and adsorption properties of the catalysts were calculated from N_2_ adsorption isotherms at 77.15 K by using a Quantachrome NOVA 1200 instrument. Before the analysis, the samples were first outgassed at 473.15 K for 2 h and cooled to 77.15 K at 1 atm (10% de N_2_/He). The specific surface area (*S*_BET_), pore volume (*V*_p_), micropore volume (*V*_mp_), and pore diameter (*D*_p_) of zeolites were calculated according to Brunauer–Emmet–Teller (BET) and Barrety–Joyner–Halenda (BJH) methods.^12,56^

Scanning electron microscopy (SEM) analyses were performed using a Zeiss EVO MA10 operated under ultrahigh vacuum conditions. The natural (NC) and modified (MC) clinoptilolite samples were carefully mounted on clean electron microscopy stubs and subsequently coated with a thin layer of gold by using a Leica EM SCD050 sputter coater. An accelerating voltage of 5.00 kV was employed, and micrographs were acquired at magnifications ranging from 0.5 to 20 kx.

### 2.3 Pyrolysis Experiments

Fixed-bed pyrolysis experiments were conducted in a tubular bench-scaled quartz reactor (the inner diameter is 4 cm and the heated zone length is 20 cm) heated by a Fortlab FT 1200 H/V electric furnace to determine the yield and properties of bio-oil obtained from macadamia carpel under different conditions. Each experiment was performed using 10 g of sample, which was placed in the reactor and heated under a continuous nitrogen flow rate of 100 mL/min to ensure an inert atmosphere. The heating rates were varied at 10, 30, and 50 K/min, while the reaction temperatures were set at 773.15, 873.15, and 973.15 K. The residence time at the final temperature was maintained at 20 min for all experiments. The operating conditions were selected based on a thermokinetic analysis of macadamia carpel pyrolysis, determined through thermogravimetric analysis.^55^

For catalytic pyrolysis, natural (NC) and MC catalysts were physically mixed with the biomass prior to pyrolysis at concentrations of 0, 10, and 20 wt %. These concentrations were selected to investigate the effect of catalyst loading on bio-oil yield, chemical composition, and deoxygenation efficiency, while optimizing the process efficiency in terms of cost-effectiveness and catalytic performance. The 0% concentration served as a control to evaluate the effects of thermal pyrolysis in the absence of catalytic activity. The 10% and 20% concentrations are chosen to examine the incremental impact of the catalyst. The condensable vapors were collected by using a series of two glass condensers submerged in an ice bath, and the liquid yield was determined gravimetrically. After each experiment, the solid char produced was weighed, while the gaseous yield was estimated by the difference from the total mass balance. A schematic representation of the experimental setup is provided in Figure 1.

**Figure 1:**
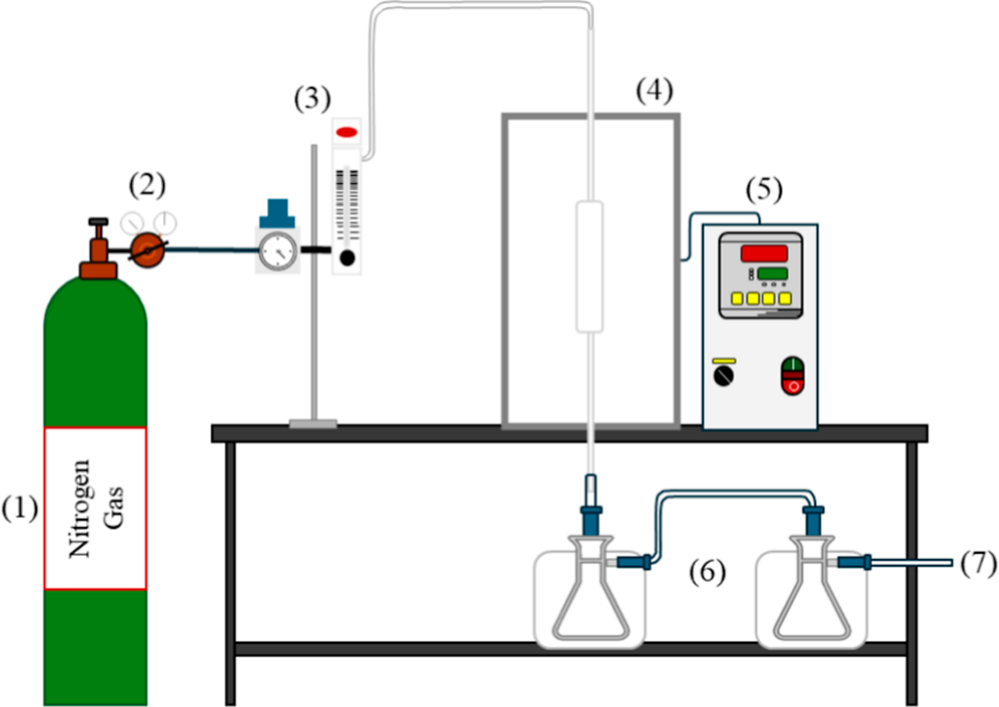
Schematic diagram of the quartz fixed-bed reactor used in this study.

The pyrolysis diagram is composed of a nitrogen cylinder (1), a valve (2), the mass flowmeter (3), the quartz reactor installed in the furnace (4), a temperature controller (5), an ice-cooled bath system (6), and the noncondensable gases exit (7).

In this research, three unreplicated 2-level full factorial designs of experiments (DoE) were conducted to optimize the pyrolysis process and to evaluate the effect of pyrolysis conditions on bio-oil production from macadamia carpel. Only one biomass (macadamia carpel) was used, but two catalytic scenarios were investigated: noncatalytic pyrolysis and catalytic pyrolysis using either NC or MC. For design (a), pyrolysis of macadamia carpel without catalyst, a 2^2^ factorial design (2 variables at 2 levels) with 3 replicates at the center point was employed, resulting in a total of 7 experiments. For designs (b) and (c), catalytic pyrolysis using NC and MC as catalysts, respectively, a 2^3^ factorial design (3 variables at 2 levels) with 3 replicates at the center point was performed, resulting in 11 experiments each. The responses measured include the yields of solid (*Y*_S_), liquid (*Y*_L_), and gaseous (*Y*_G_) products, and the data were statistically analyzed to assess the influence of the temperature (*T*), heating rate (β), and catalyst concentration (*C*) on bio-oil production. All statistical analyses were performed using TIBCO Statistica (version 14.1.0—2023). Table 2 details the design parameters.

**Table 2:** Factors and Levels for the Factorial Designs

		level		
factor		–1	0	+1
*T*	temperature (K)	773.15	873.15	973.15
β	heating rate (K min^–1^)	10	30	50
*C*	catalyst concentration (wt %)	0	10	20

The optimal conditions for liquid production were determined using the desirability function approach. This optimization method converts each of the estimated response variables into an individual desirability function *D*, which varies over the range of 0–1. Hence, the input parameters that maximize the liquid yield are found, and as long as the overall desirabilities are close to 1, the predicted values are close to the experimental ones.^52,60,61^

### 2.4 Bio-Oil GC–MS Analysis

Bio-oil was separated from the liquid product by adding 40 g of deionized water to 5 g of each sample. The mixture was centrifuged at 6000 rpm and room temperature for 10 min, when it was possible to observe the division between the polar (water-soluble) and nonpolar phases (bio-oil–water-insoluble). The bio-oil was then decanted and dissolved in dichloromethane solvent (PanReac Applichem, 99%) in a proportion of 0.01 g of oil to 10 mL of solvent. Therefore, the solutions passed through a syringe filter (0.22 μm) and injected into a GC–MS.^38,62^

The components of the obtained liquid products were analyzed using a GC-MS-QP 2010 instrument (Shimadzu) with a flame ionization detector. The GC conditions, column oven temperature progress, column used, and MS conditions are shown in Table 3.

**Table 3:** GC–MS Conditions

GC conditions	
column oven temperature	343.15 K
injection mode	split
injection temperature	473.15 K
split ratio	10
flow control mode	linear velocity
column flow	1.51 mL min^–1^
carrier gas	helium 99.9995% purity

column oven temperature progress		
rate	temperature (K)	hold time (min)
	343.15	2.0
10	573.15	7.0
		(32 min total)

column: DB-5	
length	30.0 m
diameter	0.25 mm
film thickness	0.25 μm

MS conditions	
ion source temperature	473.15 K
interface temperature	513.15 K
start *m*/*z*	40
end *m*/*z*	1000

## 3 Results and Discussion

### 3.1 Characterization of Catalysts

The XRD patterns of the catalysts and standard IZA clinoptilolite are presented in Figure 2. The main typical clinoptilolite intensities occur at 9.90°, 11.18°, 22.50°, and 30.00° and indicate the mineral is connected with Na, Ca, and K,^63,64^ while smaller peaks at 15° and 24° represent Na bounds. In comparison with the standard, NC patterns showed intense characteristic peaks of clinoptilolite at 2θ values of 10.16°, 11.5°, 13.06°, 21.98°, 22.68°, 23.74°, 28.36°, and 30.32°,^44,65,66^ which are in agreement with those of clinoptilolite IZA (Na,K,Ca_0,5_)_6_[Al_6_Si_30_O_72_]·20H_2_O).

**Figure 2:**
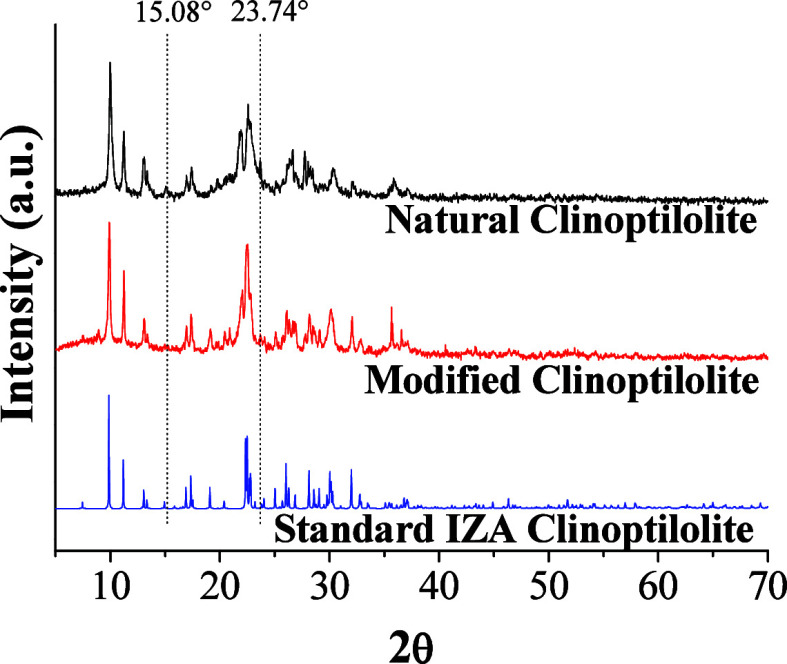
XRD patterns for the catalysts.

MC patterns are similar to the standard, which means the thermal degradation did not affect the crystalline structure.^66,67^ However, the patterns did not show intense peaks at 13.06°, 21.98°, and 22.68°, which correspond to smaller contents of Na, Ca, and K linked to the zeolite. Also, in comparison to the NC patterns, the peaks at 15.08° and 23.74° disappeared, which proves the Na removal by the activation proceeding.^67^

The calculated mean crystallite size for NC was approximately 11.94 nm, while MC exhibited a mean crystallite size of 13.06 nm. These results indicate that the modification process, which involves ion exchange and calcination, does not significantly alter the overall crystalline domain dimensions.

The pH measurements indicate that NC exhibits a pH of 6.19 ± 0.03, whereas MC shows a significantly lower pH of 4.52 ± 0.02. These findings are in agreement with the reported by Gurevich Messina et al.,^34^ who observed a pH of approximately 6.0 for NC (CL) and 4.3 for the protonated clinoptilolite (Z2). According to the author, this substantial decrease in pH following modification is indicative of the increased concentration of Brønsted acid sites, which arises from the ion exchange process where Na^+^ and other alkaline cations are replaced by NH_4_^+^ and subsequently converted to H^+^ during calcination.

FTIR spectra for NC and MC were broadly similar, as shown in Figure 3. The absorption at 3700–2350 cm^–1^ in both zeolites represents the O–H stretching vibration of hydroxyl, indicating the presence of water in a minimum amount. These bands, together with the band observed at 1617 cm^–1^ (O–H bending), typically indicate water molecules associated with Na^+^ and/or Ca^+^ in the zeolite channels.^68,69^

**Figure 3:**
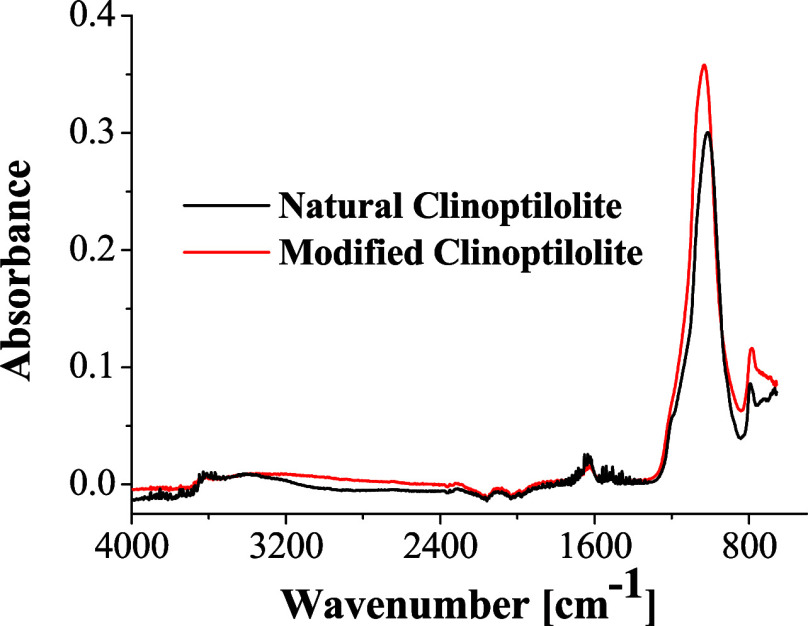
FTIR spectra of the catalysts.

Notably, in the MC, the 1617 cm^–1^ band is significantly more intense than in NC. This enhancement is attributed to the removal of Na^+^ ions via ion exchange and subsequent protonation of the zeolite, which increases the concentration of Brønsted acid sites.^70^ This observation is further supported by pH measurements, which confirm the increased acidity. The resulting higher acidity plays a crucial role in promoting deoxygenation and cracking reactions during pyrolysis, thereby enhancing catalytic performance and yielding a higher-quality bio-oil.^71^

The intensity registered at the 1028 cm^–1^ band, assigned to asymmetric T–O–T stretching vibration (T = Si or Al atoms), remains prominent in both NC and MC, indicating that the overall zeolite structure is preserved despite ion exchange.^72^ The peak at 808 cm^–1^ could be attributed to Al–O–Si bonds, which further confirms the structural integrity of these minerals.^72,73^ The retention of this band underscores the structural integrity of the zeolite remains intact after Na^+^ removal.

N_2_ adsorption–desorption isotherms for the natural and activated catalysts are illustrated in Figure 4. It could be seen that the behavior exhibited in both isotherms presented typical characteristics of isotherms type IV, because of their shape, according to IUPAC classification.^74^

**Figure 4:**
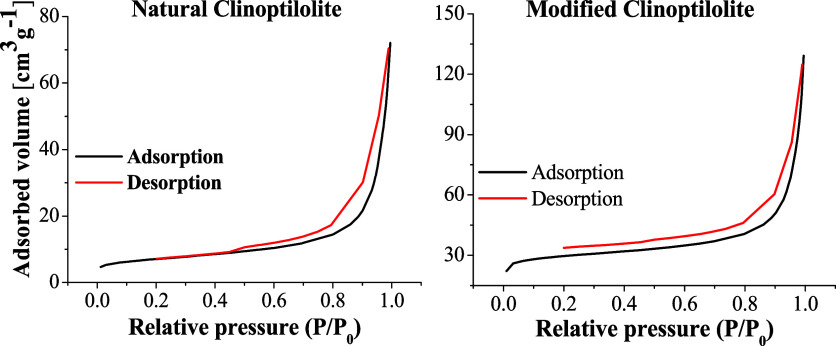
N_2_ adsorption–desorption isotherms for the natural and MC.

These kinds of shapes represent mesoporous materials, wherein there is a great adsorption at the beginning due to the strong interaction between the first gas molecules and the most active sites of the mineral. The adsorption is lower at intermediate relative pressures when the gas interacts with lower energy sites. At the end, there is a fast increase of adsorbed N_2_, which is characterized by the formation of multiple layers of gas on the surface, followed by condensation.^75,76^

The results indicate that after the acid treatment, the mineral presented a bigger amount of gas adsorbed at the beginning. Thus, the activation of clinoptilolite increased its adsorption capacity, which contributes to a better catalytic performance in pyrolysis because of the amount of condensable gases adsorption.^34^ The textural properties evaluated from the isotherms are summarized in Table 4.

**Table 4:** NC and MC Textural Properties

catalyst	*S*_BET_ (m^2^ g^–1^)	*V*_p_ (m^3^ g^–1^)	*V*_mp_ (m^3^ g^–1^)	*D*_p_ (nm)
NC	24.99	0.088	0.003	9.052
MC	101.11	0.135	0.030	10.665

The results indicate that both *S*_BET_ and the pore size distribution notably increased after the clinoptilolite modification. The MC textural values are much higher than the NC values, which confirms the zeolite activation by the ion exchange process.^76^ The *S*_BET_ strongly increased, implying that the modified sites are more accessible to the vapors/reagents, where the catalytic cracking occurs.^34,77^

The *V*_p_ and *V*_mp_, which are connected to the pore size distribution, also increased after the catalyst activation, explaining the higher N_2_ adsorption at the beginning of MC curve (Figure 4) and improving the mineral catalytic performance.^34,76^ Large pore diameters facilitate the molecules transportation, and this property is directly related to the mineral selectivity.^72,75^ There was a slight increase of *D*_p_, indicating the zeolite can allow bigger molecules passage, although both average values are interesting specially for catalytic cracking of heavy oil.^72^

The SEM micrographs of both NC and MC (Figure 5) exhibit materials that are composed of aggregated plate-like structures of varying dimensions. No significant alterations in the overall morphology were observed following the ion exchange and thermal treatment, indicating that these modification processes did not induce drastic alterations in the material’s morphology. This observation agrees with that reported in ref 34 and the calculated mean crystallite size. However, the MC micrographs exhibit more pronounced and widened pores compared to NC, probably resulting from the leaching of exchangeable cations (e.g., Na^+^ and Ca^2+^) during acid treatment.

**Figure 5:**
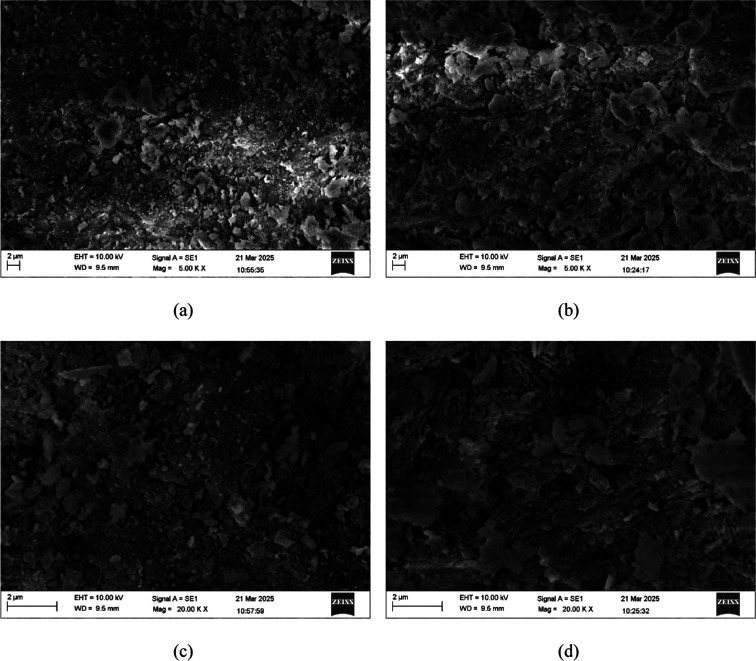
SEM micrographs of NC at 5000 (a) and 20,000 (c) and MC at 5000 (b) and 20,000 (d).

### 3.2 Pyrolysis Experimental Results and Variable Effects

#### 3.2.1 Macadamia Carpel Pyrolysis (a)

The results from the experimental design of macadamia carpel pyrolysis (a) are presented in Table 5, and its analysis shows that in the absence of a catalyst, there were no great variations on *Y*_L_ for different temperatures or heating rates, in general.

**Table 5:** Design Matrix and Experimental Data of the Obtained Yields from the Non-Catalytic Design (a)

	factors		yield (wt %)		
run	*T* (K)	β (K min^–1^)	*Y*_S_	*Y*_L_	*Y*_G_
1	973.15	50	29.90	45.90	24.20
2	773.15	50	37.71	45.77	16.52
3	973.15	10	31.07	45.85	23.08
4	773.15	10	38.30	45.10	16.60
5	873.15	30	29.41	48.16	22.43
6	873.15	30	30.12	48.69	21.18
7	873.15	30	29.71	48.52	21.73

However, it is noteworthy that the liquid fraction (*Y*_L_) was consistently higher than those of the solid (*Y*_S_) and gaseous (*Y*_G_) fractions. This predominance can be attributed to the high volatile matter content of the macadamia carpel (72.40 ± 1.21), which favors the generation of condensable vapors that subsequently form bio-oil upon cooling.^30^ Moreover, the yields of biomass pyrolysis products are strongly influenced by reaction conditions, such as heating rate and maximum pyrolysis temperature, with the highest liquid yields typically obtained at moderate temperatures (673–873 K) and heating rates (<100 K min^–1^).^14^ Under these favorable conditions, the thermal decomposition process achieves an optimal balance between primary bond-breaking reactions with secondary vapor-phase cracking, thereby maximizing condensable organic compounds.^14^

At higher temperatures, the gas production increased while the solid decreased, which occurs due to the secondary reactions that happen at high temperatures.^78^ Lower temperatures and heating rates further the occurrence of carbonization, which is responsible for a bigger solid production^79^ that can be observed in the *Y*_s_ values.

The effect of the input parameters and their interactions on fitted model can be determined by the *p*-value. The confidence interval used was 95%, thus, a low *p*-value (<0.05) represents that the variable is statistically significant. The effects of the curvature, *T* and β on the *Y*_L_ result are summarized in Table 6.

**Table 6:** Effect of Independent Factors on Yield of Liquid Product from Design (a) with *R* ^2^ = 0.99

factor	effect	standard error	*t* (2)	*p*-value	–95%	+95%
mean/interception	45.66	014	337.42	0.00	45.07	46.24
curvature	5.60	0.41	13.56	0.01	3.83	7.38
*T*	0.44	0.27	1.63	0.25	–0.72	1.60
β	0.36	0.27	1.33	0.32	–0.80	1.52
*T*/β	–0.31	0.27	–1.15	0.37	–1.47	0.85

It was found that the value of *R* ^2^ for the *Y*_L_ model was >0.95, which indicates that the experimental responses agree with the predicted values.^54^ It is observed that the curvature significantly affected the liquid yield for the macadamia carpel pyrolysis (a), representing that the linear model used may not be the best model for these experiments, although there is great adjustment. Also, neither variables nor their interaction significantly affected liquid production.

#### 3.2.2 Macadamia Carpel Pyrolysis Catalyzed by NC (b)

The results obtained from design (b) are shown in Table 7, where it is possible to observe that the maximum *Y*_L_ obtained was 52.37% at higher *T* and β. At the same conditions in the design (a), the *Y*_L_ was 45.90% (Table 5), which demonstrates that the presence of NC itself was enough to increase the liquid yield, and, in general, it also reduced the gas production, which aligns with findings reported in the literature.^46,47,80^ The pH measurements confirm that NC has a more acidic character (pH = 6.19) compared to the pyrolysis environment without a catalyst, which helps promote thermal cracking and limits the formation of solid residues. Although NC has a lower acidity compared with MC, it still exhibits a significant concentration of acid sites capable of facilitating the cleavage of C–O and C–C bonds in pyrolysis vapors. This leads to an increase in the formation of condensable vapors, thereby enhancing the liquid yield.

**Table 7:** Design Matrix and Experimental Data of the Obtained Yields from the Catalytic Design (b)

	factors			yield (wt %)		
run	*T* (K)	β (K min^–1^)	*C* (%)	*Y*_S_	*Y*_L_	*Y*_G_
1	973.15	50	20	28.65	52.37	18.98
2	773.15	50	20	48.84	44.24	7.02
3	973.15	50	0	29.90	45.90	24.20
4	773.15	50	0	37.71	45.77	16.52
5	973.15	10	20	27.02	50.08	22.90
6	773.15	10	20	44.17	43.92	11.91
7	973.15	10	0	31.07	45.85	23.08
8	773.15	10	0	38.30	45.10	16.60
9	873.15	30	10	27.76	52.09	19.32
10	873.15	30	10	31.17	52.09	16.74
11	873.15	30	10	31.55	51.55	16.91

The increased liquid yield can be attributed to the catalytic properties of NC, as evidenced by catalyst characterization analyses (Section 3.1). pH measurements confirm that NC exhibits a more acidic character (pH = 6.19) compared to the pyrolysis environment without a catalyst, which contributes to promoting thermal cracking and limiting the formation of solid residues. Although NC has a lower acidity than MC, it still possesses a sufficient concentration of Brønsted acid sites capable of facilitating the cleavage of C–O and C–C bonds in pyrolysis vapors. This catalytic activity promotes the generation of condensable volatiles, thereby increasing bio-oil production.^40–43^ Furthermore, the catalytic effect of NC in macadamia carpel pyrolysis demonstrates a comparable or even superior performance when compared to other biomass pyrolysis processes, such as switchgrass pyrolysis catalyzed by the same material, which yielded approximately 49% liquid fraction.^47^

The effects of the curvature, the variables *T*, β, and *C,* and their respective interactions on *Y*_L_ for design (b) are shown in Table 8, whose model value of *R* ^2^ was also >0.95, indicating that the experimental responses agree with the predicted values.^54^ Also, the curvature showed a significant effect, representing that the linear model used may not be the best model for these experiments, although it had great adjustment.

**Table 8:** Effect of Independent Factors on the Yield of the Liquid Product from Design (b) with *R* ^2^ = 0.98

factor	effect	standard error	*t* (2)	*p*-value	–95%	+95%
mean/interception	46.65	0.11	423.25	0.00	46.18	47.13
curvature	10.51	0.42	24.90	0.00	8.70	12.33
*T*	3.79	0.22	17.20	0.00	2.84	4.74
*C*	2.00	0.22	9.06	0.01	1.05	2.95
β	0.83	0.22	3.78	0.06	–0.12	1.78
*T*/*C*	3.35	0.22	15.21	0.00	2.40	4.30
*T*/β	0.34	0.22	1.53	0.27	–0.61	1.29
*C*/β	0.47	0.22	2.14	0.17	–0.48	1.42

The temperature is one of the most significant factors and directly affected the *Y*_L_ on design (b) as well as the catalyst concentration (*C*) and these respective parameters interaction. This occurs because the increasing temperature accelerated the volatile production, resulting in a higher gaseous and liquid yield.^15^ Moreover, the NC acts hydrolyzing biomass molecules, which explains the increasing biomass devolatilization.^39^

#### 3.2.3 Macadamia Carpel Pyrolysis Catalyzed by MC (c)

The results for design (c) are summarized in Table 9. In comparison with the other designs (a and b), macadamia carpel pyrolysis catalyzed by MC exhibited the best performance, presenting a maximum liquid yield of 58.20%.

**Table 9:** Design Matrix and Experimental Data of the Obtained Yields from the Catalytic Design (c)

	factors			yield (wt %)		
run	*T* (K)	β (K min^–1^)	*C* (%)	*Y*_S_	*Y*_L_	*Y*_G_
1	973.15	50	20	29.89	58.20	11.91
2	773.15	50	20	34.92	53.33	11.75
3	973.15	50	0	29.90	45.90	24.20
4	773.15	50	0	37.71	45.77	16.52
5	973.15	10	20	22.26	45.77	31.97
6	773.15	10	20	27.65	46.95	25.40
7	973.15	10	0	31.07	45.85	23.08
8	773.15	10	0	38.30	45.10	16.60
9	873.15	30	10	29.17	52.12	18.71
10	873.15	30	10	28.90	50.14	20.96
11	873.15	30	10	28.78	50.84	20.38

The MC catalytic effect is shown to be more effective than the NC effect, which can be explained by the formation of stronger active sites in the crystalline structure after modification.^46^ As demonstrated by the comprehensive characterization of catalysts (Section 3.1), the MC exhibits enhanced catalytic properties compared to NC. XRD, FTIR, and pH measurements confirm the removal of Na^+^ and the subsequent increase in acidity, while BET analysis and SEM images reinforce that the surface area and pore volume of MC are substantially higher than those of NC. Besides, for all the runs, the biggest yield is the *Y*_L_ and, in general, the *Y*_G_ increased in comparison to the other designs of experiments.

These modifications in the MC directly influence product distribution during pyrolysis. The enhanced Brønsted acidity and improved porosity facilitate the diffusion of reactants and promote efficient deoxygenation and cracking reactions, resulting in a higher liquid yield (*Y*_L_) and increased gas yield (*Y*_G_) compared to noncatalytic and NC-catalyzed experiments. Specifically, the enhanced acid sites in MC accelerate the cleavage of C–O bonds, leading to more efficient oxygen removal from bio-oil precursors. This process generates additional CO and CO_2_, which contribute to the observed gas yield.^81^

The effects of the curvature, the factors *T*, β, and *C,* and their respective interactions on *Y*_L_ for design (c) are shown in Table 10, whose model value of *R* ^2^ was also >0.95; thus ,the experimental responses agree with the predicted values.^54^

**Table 10:** Effect of Independent Factors on the Yield of the Liquid Product from Design (c) with *R* ^2^ = 0.96

factor	effect	standard error	*t* (2)	*p*-value	–95%	+95%
mean/interception	48.36	0.36	136.23	0.00	46.83	49.89
curvature	5.35	1.36	3.94	0.06	–0.50	11.20
*T*	1.14	0.71	1.61	0.25	–1.91	4.20
*C*	5.41	0.71	7.62	0.02	2.35	8.46
β	4.88	0.71	6.88	0.02	1.83	7.94
*T*/*C*	0.70	0.71	0.99	0.43	–2.35	3.76
*T*/β	1.36	0.71	1.91	0.20	–1.70	4.41
*C*/β	4.52	0.71	6.37	0.02	1.47	7.58

The curvature did not have a significant effect in this design, so that the linear model can represent these experiments (design c) very well. The effects of *C* and β were significant, as well as their interactions (*C*/β), and directly increased the liquid yield. The clinoptilolite modification intensified this mineral hydrolysis capacity and, for this reason, the MC concentration became the most significant parameter.^11,44,62^

### 3.3 Optimization

The factors *T*, β, and *C* were simultaneously optimized by using the desirability function approach, in order to maximize the *Y*_L_ and minimize the *Y*_S_ and *Y*_G_ for the three different designs.

The optimum values of *T* and β were obtained through the desirability analysis for macadamia carpel pyrolysis (a), which is shown in Figure 6. The overall desirability obtained was 0.99 (very close to 1), which indicates that the predicted optimized results are in accord with the experimental output. The desirability was obtained at 873.15 and 50 K min^–1^, with a liquid percentage of 48.64%. In comparison with the experimental results, the maximum *Y*_L_ obtained in design (a) was 48.69% (Table 5) at 873.15 and 30 K min^–1^, confirming that these optimum values are very close to the experimental results.

**Figure 6:**
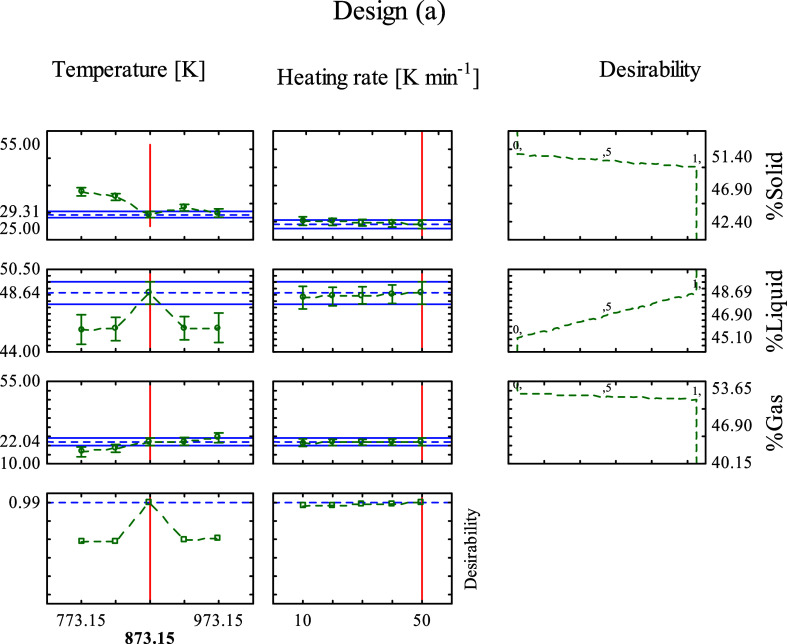
Desirability analysis for the optimization of input variables to maximize the liquid yield produced by the macadamia carpel pyrolysis, design (a).

The desirability analysis of the optimum values of *T*, β, and *C* for the macadamia carpel pyrolysis catalyzed by NC (b) is shown in Figure 7. The overall desirability was 0.77 (near to 1), which also indicates an agreement of the predicted optimized results with the experimental output. It was obtained at 873.15 and 50 K min^–1^, with 20% of catalyst, resulting in an estimated liquid percentage of 53.76%. The maximum experimental *Y*_L_ was 52.37% (Table 7), which was got at 973.15 and 50 K min^–1^, with 20% of catalyst. The predicted and experimental values are also very close to each other, as well as their conditions, which agree with the desirability.

**Figure 7:**
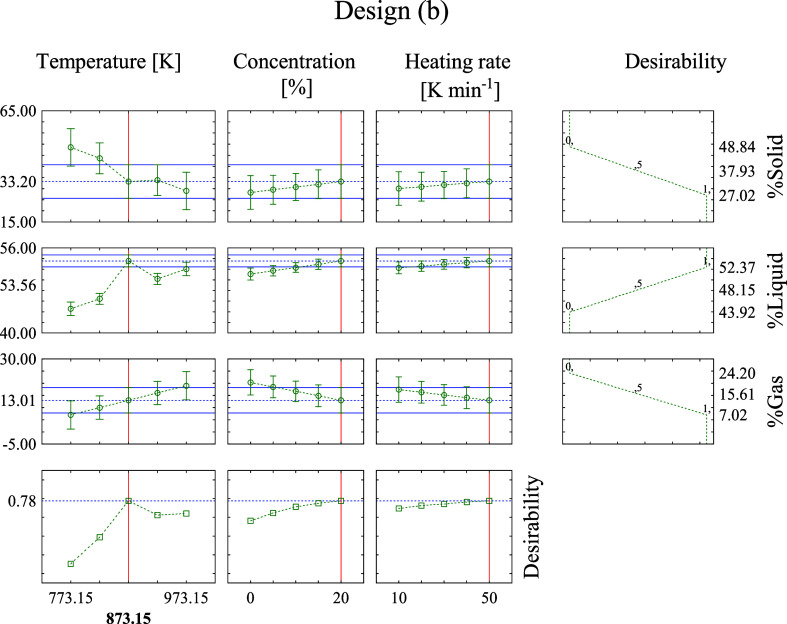
Desirability analysis for the optimization of input variables to maximize the liquid yield produced by the macadamia carpel pyrolysis catalyzed by NC, design (b).

The macadamia carpel pyrolysis catalyzed by MC (c) optimum conditions is represented in Figure 8. The overall desirability found was 0.81 (close to 1), indicating an agreement of the predicted optimized results with the experimental output again. This desirability was found at 873.15 and 50 K min^–1^, with 20% of catalyst, which resulted in an estimated liquid percentage of 58.44%. The maximum experimental *Y*_L_ was 58.20% (Table 9), and it was obtained at 973.15 and 50 K min^–1^, with 20% of catalyst. One more time the predicted and experimental values and respective conditions are very similar, which agree with the obtained desirability.

**Figure 8:**
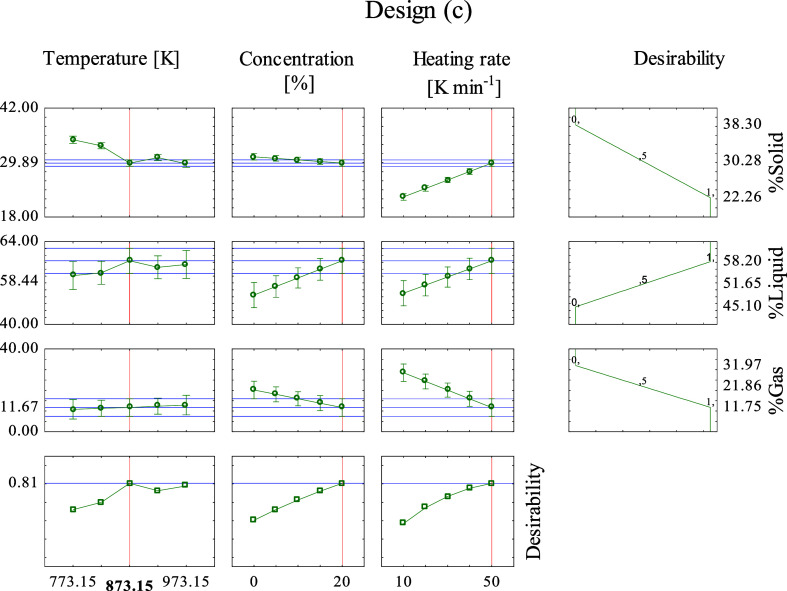
Desirability analysis for the optimization of input variables to maximize the liquid yield produced by the macadamia carpel pyrolysis catalyzed by MC, design (c).

It can be observed that for all designs (a, b, and c), the optimum temperatures and heating rates were the same (873.15 and 50 K min^–1^), and at catalytic pyrolysis designs, the optimum catalyst concentration was 20%. Further, the highest estimated percentage of liquid was obtained using the MC, which agrees with the experimental obtained results.

The pyrolysis experimental results clearly indicate that the catalytic performance improves with zeolite modification (MC > NC > no catalyst). However, the cost implications of using modified zeolites (MC) should be considered. Modifying zeolites typically involves additional processes, which can significantly increase the material costs compared to using unmodified zeolites or no catalyst. While modified zeolites enhance the liquid yield, the cost implications must be carefully weighed to achieve optimal economic outcomes in macadamia carpel pyrolysis processes.

Thus, decisions regarding catalyst selection should consider both performance and economic factors to ensure optimal process efficiency and profitability. When assessing the process’s cost-effectiveness, it is essential to consider not only the production costs of the catalysts but also the economic benefits from improved bio-oil quality. Phenols, alkanes, and reduced acids significantly enhance the bio-oil’s value as a fuel and in industrial applications. Therefore, despite the initial costs associated with catalyst modification, the improvement in bio-oil quality may justify the investment in zeolites by increasing the overall product value and market competitiveness.

### 3.4 GC–MS of Bio-Oil Samples

The GC–MS analysis of the bio-oil samples produced from macadamia carpel pyrolysis indicated a great number of chemical compounds. Usually, the bio-oil from pyrolysis of lignocellulosic biomass contains more than 300 compounds,^82^ so for this reason, only the compounds with a peak match greater than 80% and an area greater than 1% were considered in this study.

The compounds were also classified according to their organic groups in Tables 11–13, according to the respective DoE (a, b and c), which demonstrated that phenols, alkanes, carboxylic acids, and ethers were major components found in bio-oil samples in general.

**Table 11:** Major Compounds Identified in Bio-Oils (Area, %) from Macadamia Carpel Pyrolysis (a) at Different Reaction Conditions Detected Using GC–MS

heating rate (K min^–1^)	10		50	
compound/temperature (K)	773.15	973.15	773.15	973.15
acids		6.38		9.16
alcohols	15.09	3.59		
alkanes	5.61		17.67	
ethers			38.59	
furans		6.83		
phenols	79.30	83.20	43.74	90.84

**12:** Major Compounds Identified in Bio-Oils (Area, %) from Macadamia Carpel Pyrolysis Catalyzed by 20% of NC (b) at Different Reaction Conditions Detected Using GC–MS

heating rate (K min^–1^)	10		50	
compound/temperature (K)	773.15	973.15	773.15	973.15
acetones			12.07	5.55
acids	28.20		1.84	28.59
alcohols				39.47
aldehydes				4.73
alkanes		43.66	7.21	13.76
Ethers	21.31		4.82	1.81
Furans			1.68	
phenols	50.49	56.34	72.38	6.09

**Table 13:** Major Compounds Identified in Bio-Oils (Area, %) from Macadamia Carpel Pyrolysis Catalyzed by 20% of MC (c) at Different Reaction Conditions Detected Using GC–MS

heating rate (K min^–1^)	10		50	
compound/temperature(K)	773.15	973.15	773.15	973.15
acetones		6.94	8.65	4.65
acids		5.48		1.85
alkanes		7.40		
ethers		9.44	6.44	7.39
furans			10.12	5.09
phenols	100.00	70.74	74.79	81.02

For all the designs, the major components identified were phenols, which primarily come from lignin decomposition and is directly related to the biomass feedstock lignocellulosic composition.^83^ These compounds are considerably interesting in biomass refineries^84^ and the production of renewable phenols from biomass is used to replace fossil phenols, besides phenols and its derivatives are valuable for the resin and adhesive industry.^39^ This attractive application of phenols encouraged many researches that use catalysts, such as zeolites, to enhance the yield and select specific phenols.^85^

It is possible to observe that the phenol content obtained from all designs was higher than 70%. However, the biggest phenol amounts (approximately 100.00%) were produced from the pyrolysis catalyzed by MC, which occurred at 773.15 and 10 K min^–1^. It indicates that this catalyst can be used to favor phenol production, especially because of the financially favorable reaction conditions at lower temperatures and heating rates.

The experiments without a catalyst also produced a high content of phenols (90.84%), but at high temperatures and heating rates (973.15 and 50 K min^–1^), which is not preferable from the operational point of view. The bio-oil from pyrolysis with NC showed less phenol amounts than the other designs, since the highest content of phenols was 72.38%, and it was found at 773.15 and 50 K min^–1^. Thus, besides the phenol contents being the lowest, the variable values are not financially worth it, in comparison with the results obtained by design (c).

The presence of alkanes in bio-oil increases its quality as a fuel^86^ and the highest percentages of alkanes were obtained in catalytic pyrolysis in the presence of NC. The corresponding reaction conditions occurred at high temperature (973.15 K) and low heating rate (10 K min^–1^), resulting in 43.66% of alkanes. Hence, it indicates that the use of NC as a catalyst favors alkane production and, consequently, the bio-oil fuel properties.

In the presence of MC, the biggest alkane percentage was 7.40%, obtained at 973.15 and 10 K min^–1^, and in absence of a catalyst, the highest alkane content was 17.67% at 773.15 and 50 K min^–1^. So, neither design (a) nor design (c) reached the alkane content from design (b) and its operational conditions.

Furans are considered stable compounds that increase the fuel energy value^39^ and were most obtained in the experiments with MC (10.12% at 773.15 and 50 K min^–1^). Alcohols are valuable compounds^39,73^ mostly found in the presence of NC (39.47%) at high temperature and heating rates.

Oxygenated compounds are considered to be undesirable fractions. High oxygen content in bio-oil markedly reduces its energy efficiency by lowering the heating value. Oxygenated compounds such as carboxylic acids, alcohols, aldehydes, and ethers possess lower carbon-to-hydrogen ratios than conventional hydrocarbons, thus diminishing the energy density of the bio-oil.^33,34^ The compatibility with the existing fuel infrastructure is equally constrained by the bio-oil oxygen content. Elevated levels of oxygenates increase viscosity and molecular weight due to their tendency to undergo polymerization during storage and processing, thereby complicating both handling and combustion processes.^34^ Additionally, the polar nature of these compounds also renders bio-oils immiscible with hydrocarbon fuels and corrosive to storage systems directly affecting its stability^39^ and its compatibility with existing fuel infrastructure.^50^

In general, the biggest amounts of acids were found at high temperatures and heating rates, and the bio-oils from pyrolysis catalyzed by NC (design b) showed the greatest amount (28.59%) at 973.15 and 50 K min^–1^. At the same conditions, the pyrolysis catalyzed by MC and the pyrolysis in the absence of a catalyst produced 1.85% and 9.16% of acids, respectively, indicating that the use of MC can reduce the acids amounts.

Other compounds that are common in bio-oil were also obtained, even in less amounts, from the designs of experiments such as aldehydes and ethers, oxygenated compounds that are undesirable.^85^ These displeasing compounds were usually found at high temperatures and heating rates, albeit in lower amounts compared to other components, and, in general, their concentration decreased in the presence of the catalysts.

The enhanced catalytic performance observed with MC is closely linked to its modified physicochemical characteristics, as demonstrated by catalyst characterization results (Section 3.1 Characterization of Catalysts). The XRD analysis indicates that while both NC and MC retain the characteristic crystalline structure of clinoptilolite, MC exhibits reduced intensities in peaks corresponding to Na^+^, confirming the effective removal of these cations during the ion exchange process.^67^ This alteration is further corroborated by the FTIR spectra, where the MC sample shows a significantly more intense band at 1617 cm^–1^ (indicative of increased Brønsted acid sites) compared with NC. Additionally, pH measurements reveal a substantial decrease in pH from 6.19 in NC to 4.52 in MC, supporting the notion of enhanced acidity. Furthermore, BET analysis shows a considerable increase in the surface area (from 24.99 m^2^/g in NC to 101.11 m^2^/g in MC), which, along with the SEM observations of widened pores, indicates improved textural properties. Collectively, these improved modifications facilitate the diffusion of reactants and promote efficient deoxygenation and cracking reactions during pyrolysis.

Moreover, the enhanced Brønsted acidity and tailored porosity of the MC can be associated with a notable reduction in oxygenated compounds, as these characteristics collectively favor deoxygenation pathways (e.g., dehydration, decarboxylation, and decarbonylation) over condensation reactions.^34^ By removing Na^+^ through ion exchange, the catalyst minimizes unwanted neutralization effects, while the newly generated H^+^ sites selectively cleave C–O bonds in oxygenated intermediates.^49,50^ During catalytic pyrolysis, the macromolecules of cellulose, hemicellulose, and lignin break down into intermediate products—such as carboxylic acids, phenols, anhydrosugars, and light gases (CO, CO_2_, CH_4_, H_2_)—which then interact with the acid sites of clinoptilolite^40–43^ (Figure 9).

**Figure 9:**
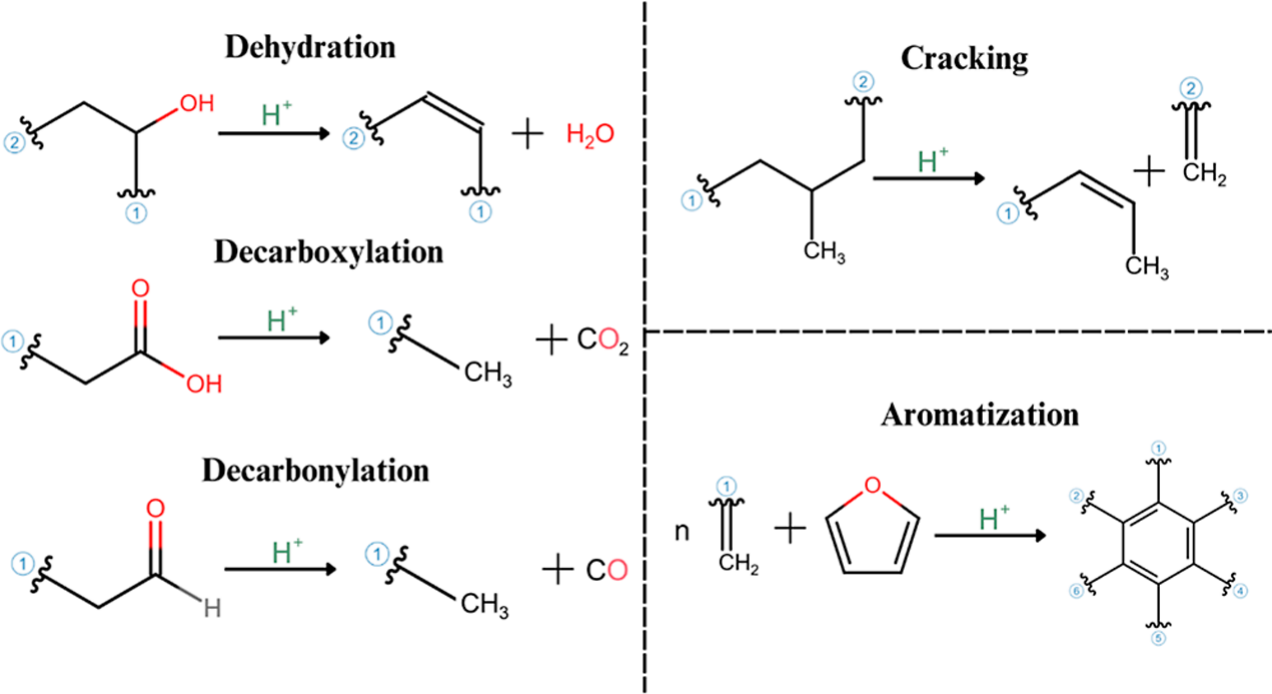
Reactions between oxygenated intermediate products and the acidic sites of clinoptilolites during heating in the absence of oxygen.

These interactions promote key reactions like deoxygenation, cracking of longer carbon chains into smaller hydrocarbons, and aromatization of oxygenated intermediates, ultimately yielding a bio-oil with lower oxygen content and improved stability.^40,42^ In contrast, NC exhibits fewer Brønsted acid sites, leading to less efficient deoxygenation. Consequently, the MC provides a more acidic environment that accelerates these reactions, further reducing oxygenated compounds in the final product.^34^

In summary, bio-oil produced without a catalyst had the lowest quality due to a higher oxygenated compound content, resulting in a lower market value despite lower costs. NC provided a balanced approach, yielding bio-oil with improved fuel properties and a higher alkane content at a moderate cost, making it viable for high-quality fuel production. MC, though more expensive due to the modification process, resulted in the highest liquid yield and bio-oil with a significant phenol content, suitable for industrial applications and fine chemical production. These results underscore the importance of considering both economic and qualitative factors in catalyst selection to optimize macadamia carpel pyrolysis for enhanced efficiency.

## 4 Conclusion

Natural and MC were characterized, and the activation reaction was verified, resulting in a higher gas adsorption capacity and better catalytic performance. Experimental designs revealed that the highest liquid yields were obtained through catalytic pyrolysis, especially with an MC (58.20%). Temperature and catalyst concentration were identified as the most significant factors directly affecting the increase in liquid percentage increase.

Desirability profiles indicated optimal input variables of 873.15 K, 50 K min^–1^, and 20% catalyst, estimating a maximum liquid yield of 58.44% for catalytic pyrolysis with MC. The curvature effect suggested that a three-level DoE is necessary to better represent the macadamia carpel pyrolysis process without a catalyst and with NC.

GC–MS analysis of the bio-oil demonstrated that the catalysts effectively reduced undesirable oxygenated compounds. Bio-oil produced with NC had quality fuel properties, while bio-oil produced with MC contained a high phenol content, which is beneficial for fine chemical production and industrial applications. Notably, phenols produced via catalytic pyrolysis with MC were obtained at lower temperatures and lower heating rates, reducing production costs.

A comprehensive cost–benefit analysis of each catalyst type (no catalyst, NC, and MC) reveals critical insights for process optimization. Using no catalyst minimizes costs but results in lower liquid yields and less desirable bio-oil quality. MC, while incurring higher costs due to additional modification processes, achieves the highest liquid yield, produces bio-oil with the highest phenol content, and reduces operational expenses. This capability can justify the higher initial investment by enhancing the overall product value and market competitiveness. Similarly, NC improves the liquid yield and bio-oil quality by increasing desirable compounds like alkanes. This improvement, coupled with moderate costs, supports NC as a cost-effective option for boosting bio-oil quality and market value.

In conclusion, decisions regarding catalyst selection should account for performance, quality, and economic factors to ensure optimal process efficiency and profitability. This comprehensive framework for evaluating the economic feasibility of using natural and modified zeolites in macadamia carpel pyrolysis ensures that catalyst selection decisions prioritize efficiency and product quality enhancement, leading to optimal process efficiency and profitability.

## Abbreviations

AC: ash content
BET: Brunauer–Emmet–Teller
BJH: Barrety–Joyner–Halenda
*D*_p_: pore diameter
DoE: design of experiments
FC: fixed carbon
FTIR: Fourier-transform infrared
GC/MS: gas chromatography/mass spectrometry
GEE: greenhouse gases emission
HHV: high heating value
*k*: Scherrer’s constant
*L*: crystalline size
M: moisture
MC: modified clinoptilolite
NC: natural clinoptilolite
*S*_BET_: surface area
SEM: scanning electron microscopy
VM: volatile matter
*V*_mp_: micropore volume
*V*_p_: pore volume
XRD: X-ray diffraction
*Y*_S_: solid yield
*Y*_L_: liquid yield
*Y*_G_: gaseous yield
λ: X-ray wavelength
τ: full width at half maximum (fwhm) of the diffraction peak
θ: Bragg angle
